# Medical Electricity

**Published:** 1893-03

**Authors:** 


*Medical Electricity.* By W. E. STEAVENSON. M.D., and H. LEWIS JONES, M.D. Pp. 446. London: H. K. LEWIS. 1892.

We feel that this book can be cordially recommended as a useful and trustworthy guide to the proper application of electricity to medical practice. It begins with a clear account of the general principles of the science, compiled with the object of giving the student that knowledge of the theory of the subject which alone will enable him to carry on his work on a sound and rational basis. Dr. Lewis Jones acknowledges the assistance of Mr. H. M. Elder in bringing this part of the work thoroughly up to date, and at the same time hopes that it may stimulate the reader to consult the more elaborate text-books on electricity. The account of the various batteries and apparatus employed in medical work is a complete one, and full of useful practical information. After an exposition of the most important facts in the physiology of the subject, the remainder of the book treats of the general and special applications of electricity to therapeutics. The statements made as to the various diseases in which advantage is gained from the employment of electricity, and the manner in which it is best carried out, leave little to be desired. The account given of the therapeutic value of statical electricity is especially interesting in view of the fact that it is comparatively so little used in this country. Chapters on electrolysis, the electro-cautery, and lighting instruments, complete the book. Dr. Jones has, so far as possible, availed himself of the manuscript left by the late Dr. Steavenson, but is himself responsible for the greater part of an excellent work. We may quote this sentence from the introduction: "A great deal of the quackery which surrounds and discredits medical electricity is due to the indifference and contemptuous attitude of the medical profession, and we have only ourselves to blame if the public insist on seeking elsewhere for treatment which is refused to them by their medical advisers."

